# Transverse colon invasion from intrahepatic cholangiocarcinoma with lymph node metastasis in the regional mesocolon: a case report

**DOI:** 10.1186/s40792-020-01010-9

**Published:** 2020-10-01

**Authors:** Kenta Aso, Kyoji Ito, Nobuyuki Takemura, Fuyuki Inagaki, Fuminori Mihara, Norihiro Kokudo

**Affiliations:** Department of Surgery, Hepato-Biliary-Pancreatic Surgery Division, National Center for Global Health and Medicine, 1-21-1 Toyama, Shinjuku-ku, Tokyo, 162-8655 Japan

**Keywords:** Intrahepatic cholangiocarcinoma, Colon invasion, Lymph node metastasis

## Abstract

**Background:**

Intrahepatic cholangiocarcinoma (ICC) is an aggressive cancer with high frequency of extrahepatic metastasis at diagnosis. However, there have been very few reports of direct invasion to transverse mesocolon with lymph node metastasis in the regional mesocolon.

**Case presentation:**

A 71-year-old man presented to our hospital with anorexia and weight loss. Abdominal computed tomography (CT) revealed enlarged gallbladder wall with intrahepatic tumor extended from the gallbladder. The transverse colon was located adjacent to the gallbladder and its wall was thickened, indicating tumor invasion. Some enlarged lymph nodes were observed in the transverse mesocolon, suggesting metastatic or inflammatory lymph node swelling. Percutaneous liver biopsy detected poorly differentiated adenocarcinoma. After confirming the absence of remote metastasis and peritoneal dissemination, surgical resection including right hepatectomy and right hemicolectomy was performed. The pathological diagnosis was adenosquamous carcinoma of the liver and lymph node metastasis in the transverse mesocolon. The surgical margins were negative and R0 resection was achieved. Although adjuvant chemotherapy was administered, follow-up CT detected multiple metastases to the lung 4 months after surgery. The patient died 12 months after the operation.

**Conclusions:**

Direct colon invasion from ICC may cause lymph node metastasis in the regional mesocolon. Careful assessment is necessary for the diagnosis of enlarged lymph nodes in ICC with direct colon invasion.

## Introduction

Intrahepatic cholangiocarcinoma (ICC) is an aggressive cancer and is sometimes diagnosed initially as a large and locally advanced tumor involving adjacent extrahepatic organs such as the diaphragm, gallbladder, or bowel [1, 2]. Although surgery is the only potentially curative treatment for patients who have resectable disease [3], there have been very few reports on surgical resection of locally advanced ICC with direct invasion to extrahepatic organs [1]. We report a case of transverse colon invasion from ICC with lymph node metastasis in the regional mesocolon.

## Case presentation

**Fig. 1:**
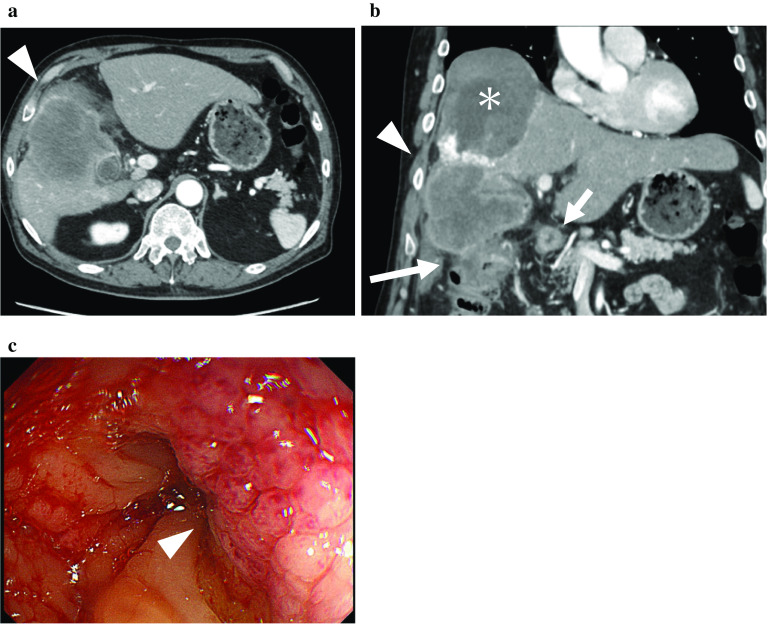
Preoperative imaging findings. **a** Preoperative abdominal computed tomography scan. Axial view (left) and coronal view (right) showed an enlarged low-attenuation mass that was 80 mm in diameter. The mass involved the S5 segment of the liver (arrowhead) and the hepatic flexure of the transverse colon (large arrow). A nodule was observed in the transverse mesocolon (small arrow). * indicates cavernous hemangioma. **b** Preoperative colonoscopy. An elevated lesion was detected (arrowhead) on the transverse colon, which was a result of compression from the tumor

A 71-year-old man with anorexia and weight loss visited our hospital in December 2018. Laboratory findings showed elevated levels of inflammatory markers and tumor markers including carcinoembryonic antigen [17.8 ng/ml (reference range: 0.0–4.9 ng/ml)], carbohydrate antigen 19–9 [765.9 U/ml (reference range: 0.0–36.9 U/ml)], and squamous cell carcinoma antigen [105 ng/ml (reference range: 0.0–1.4 ng/ml)]. Abdominal computed tomography (CT) revealed enlargement of the gallbladder, which was 80 mm in diameter and involved the S5 segment of the liver and the hepatic flexure of the transverse colon (Fig. 1a). Abdominal CT detected a nodule in the transverse mesocolon, suggesting peritoneal dissemination or lymph node swelling due to metastasis or inflammation. Colonoscopy revealed an elevated lesion on the transverse colon. Biopsy identified no malignant cells, indicating that the elevation was caused by compression from the tumor (Fig. 1b). Percutaneous liver biopsy for the diagnosis of the tumor revealed poorly differentiated adenocarcinoma.

Although gallbladder cancer with direct invasion to the liver and the transverse colon was suspected, it was difficult to confirm the diagnosis of the nodule in the transverse mesocolon and the possible diagnoses included peritoneal dissemination, lymph node metastasis, and inflammatory lymph node swelling. Therefore, the clinical stage of the gallbladder cancer was diagnosed as T4N1M1 Stage IV-B (in case of peritoneal dissemination or lymph node metastasis) or T4N1M0 Stage IV-A (in case of inflammatory lymph node swelling) according to the TNM classification of malignant tumors (eighth edition) edited by the Union for International Cancer Control [4]. Although the tumor was highly advanced, general condition of the patient was favorable and there was a possibility to achieve R0 resection. Therefore, we scheduled surgical resection including right hepatectomy, transverse colon resection, and lymph node dissection after exploring laparotomy confirmed the absence of peritoneal dissemination or remote metastasis.

**Fig. 2:**
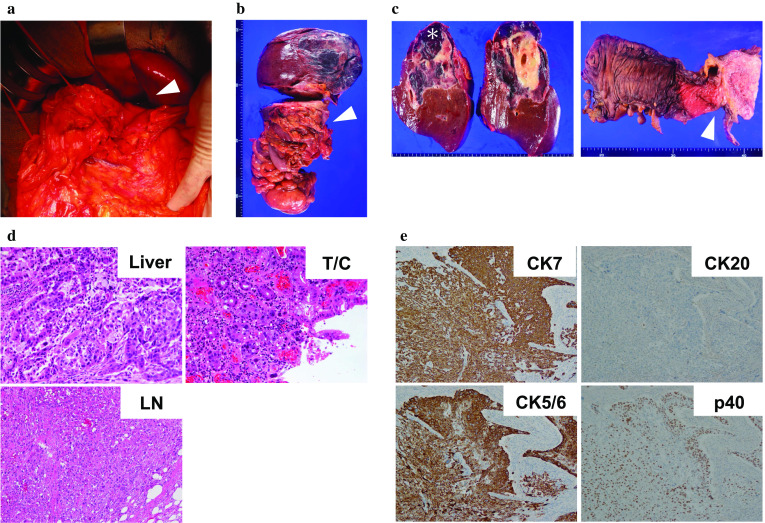
Intraoperative and pathological findings. **a** Intraoperative photograph after laparotomy. The infiltrated site of the transverse colon was taped (arrowhead). **b** The surgical specimen showed infiltration of the transverse colon (arrowhead) by the tumor. **c** Macroscopic appearance of the sections of the liver (left) and the transverse colon (right) revealed that the tumor was solitary and whitish, 8.5 × 7.5 cm in diameter, and had irregular margin. The dark-red lesion (*) was cavernous hemangioma. Direct invasion to the transverse colon was detected (arrowhead) **d** Hematoxylin–eosin staining of the tumor in the liver (Liver, × 40), transverse colon (T/C, × 40), and lymph node in the transverse mesocolon (LN, × 20) revealed glandular structures and a tendency toward keratinization. Therefore, we concluded that the tumor included an adenocarcinoma component as well as a squamous cell carcinoma component, suggesting ASC of the liver. **e** Immunostaining of the liver tumor revealed that the tumor was CK 7 (+) and CK 20 (−), indicating adenocarcinoma of the biliary system. The tumor was CK 5/6 (+) and p 40 (+), indicating that it also included a squamous cell carcinoma component (× 20)

The surgery was performed in January 2019. No ascites or peritoneal dissemination (Fig. 2a) was observed on exploring laparotomy. Intraoperative ultrasonography revealed no other intrahepatic metastases. Furthermore, there was no para-aortic lymph node swelling by macroscopic and ultrasonographic findings, and therefore, we did not conduct the sampling of para-aortic lymph nodes. The nodule detected in the transverse mesocolon was not a peritoneal dissemination, but a lymph node swelling. Thus, we proceeded with resection of the tumor. Initially, right hemicolectomy was performed and the nodule in the transverse mesocolon was simultaneously removed. Subsequently, right hemihepatectomy was performed with the clamp crushing method using the Pringle’s maneuver. The lymph nodes in the hepatoduodenal ligament were also removed. The operative time was 6 h 18 min and the estimated blood loss was 507 ml. Two units each of red blood cells and fresh frozen plasma were transfused during the operation.

Macroscopic findings of the specimen revealed a whitish mass, 8.5 × 7.5 cm in diameter, involving the transverse colon (Fig. 2b, c). Pathological analysis of the tumor demonstrated glandular structures and a tendency toward keratinization in hematoxylin–eosin staining, indicating an adenocarcinoma component and a squamous cell carcinoma component, respectively (Fig. 2d). Liver was confirmed as the origin of the tumor, since the main tumor lesion was located in the liver and there was no intraepithelial lesion in the gallbladder. The tumor invaded into the mucosal surface of the transverse colon through vascular invasion. Lymph node metastasis was identified in the hepatoduodenal ligament (2/10) and transverse mesocolon (1/3). In terms of lympho-vascular invasion, there were microscopically obvious venous invasion (v3+) and slight lymphatic invasion (ly+) in the infiltrated transverse mesocolon. The surgical margins were negative. Immunostaining of the liver tumor revealed that the tumor was cytokeratin (CK) 7 (+) and CK 20 (−), suggesting adenocarcinoma of the biliary system [5]. The tumor was CK 5/6 (+) and p 40 (+), indicating that it contained squamous epithelium [6, 7] (Fig. 2e). Consequently, the final diagnosis was adenosquamous carcinoma (ASC) of the liver [vp1, vv1, va0, sm (−), T3N1M1, Stage IV-B].

The postoperative course was uneventful except slight elevation in the level of hepatic enzymes immediately after the surgery. The patient was discharged 14 days after the surgery without major complications. Although adjuvant chemotherapy using gemcitabine (1000 mg/m^2^) and cisplatin (25 mg/m^2^) was administered, follow-up CT at 4 months after the surgery detected metastases to the lung. Although systemic chemotherapy using gemcitabine (1000 mg/m2) and S-1 (120 mg/day) was initiated, the tumor progressed gradually. The patient died 12 months after the operation.

## Discussion

ICC is the second most common primary hepatic tumor after hepatocellular carcinoma [8]. It is an aggressive cancer and is sometimes diagnosed initially as a large and locally advanced tumor involving adjacent extrahepatic organs such as the diaphragm, gallbladder, or bowel [1, 2]. Since there is no established chemotherapy regimen or alternative treatment for locally advanced ICC [1], extended surgical resection with the goal of achieving negative microscopic margins remains the cornerstone of treatment [1]. However, the prognosis is still poor with 5-year survival rate of 20–35% following surgical resection [3]. Among histological variations of ICC, ASC is a rare subtype and its incidence among cases of ICC is 2–3% [9]. ASC consists of both adenocarcinoma and squamous cell carcinoma components. Squamous differentiation is evident by individual cell keratinization, intercellular bridges, keratin pearl formation and/or dyskeratosis and glandular differentiation by various-sized gland formations and intracellular and intraluminal mucin [10]. Adenosquamous carcinoma of the liver was first reported in 1975 by Barr and Hancock [11], and 73 cases of primary liver ASC were reported by December 2016 [12]. According to the PubMed research for “adenosquamous carcinoma” and “liver”, four other cases have been published in the English-language medical literature. Regarding the features of ASC, rapid progression was specific to the ASC of the liver compared with common ICC. Furthermore, ASC of the liver spread locally and distantly in the early phase of tumor growth, and local recurrence and lymph node metastasis were likely to occur after operation. Moreover, it was presumed that this aggressive clinical course of the tumor was attributed to squamous cell carcinoma components of ASC, because squamous cell carcinoma components has more proliferating cell nuclear antigen than adenocarcinoma component, that is, growth rate of the squamous cell carcinoma component is faster than adenocarcinoma component [13, 14]. Kobayashi et al. reported a mean survival time for patients with hepatic ASC of 8.7 months, and the overall 1-, 2-, and 3-year survival rates after surgery were 38.5, 16.2, and 10.8%, respectively [15]. Furthermore, multivariate analysis by Kobayashi et al. revealed that lymph node metastasis and the elevation of total bilirubin were associated with poor survival after surgery [15].

There have been very few reports on resection of locally advanced ICC with direct invasion to extrahepatic organs. To the best of our knowledge, only two case reports in English literature by Mimatsu et al. [1] and Okada et al. [16] have reported invasion to the transverse colon similar to that observed in the present case. They reported that R0 resection of massive ICC with transverse colon invasion leads to long-term survival of the patients. In the present report, we observed a similar case of a large ICC with direct invasion to the transverse colon. We achieved R0 resection, but there was lymph node metastasis in the transverse mesocolon. Although there are few reports on “regional” lymph node metastasis from the colon directly invaded by tumor originating in other organs, the lymph node metastasis in the transverse mesocolon might have developed through the lymphatic pathways from the transverse colon invaded by ICC [17, 18].

The detailed mechanisms of regional lymph node metastasis have recently been the subject of intense interest and have been elucidated by basic research on tumor pathophysiology [19]. Briefly, tumor secretes lymphangiogenic cytokines, promoting lymphangiogenesis in the tumor. Tumor cells invade the extracellular matrix and move toward the newly developed lymphatic capillaries. Tumor cells move singly or in clusters with the lymphatic stream into the sentinel lymph node. Subsequently, they enter the node through the subcapsular sinus, invade the cortex of the lymph node, and proliferate and metastasize to the adjacent lymph nodes. According to the aforementioned mechanism, there is a possibility of “regional” lymph node metastasis from the colon invaded by tumor from other organs through lymphatic pathways. In the present case, preoperative imaging detected an enlarged lymph node in the transverse mesocolon and the lymph node swelling was diagnosed as metastasis after resection. In retrospect, another strategy such as laparoscopic sampling of the lymph node or adjuvant chemotherapy would be an alternative option, considering the early recurrence despite R0 resection. In addition, postoperative radiotherapy might be helpful to improve the prognosis of liver ASC, because the squamous cell carcinoma component may have a good sensibility for radiation [20], although further researches would be needed to prove the effectiveness of radiation therapy for ASC.

In conclusion, we performed radical surgical resection for locally advanced ICC with direct transverse colon invasion. Careful assessment of lymph node swellings in the mesocolon would be required in cases of direct colon invasion from ICC.

## Data Availability

The datasets analyzed during the present case report are not publicly available due to information that could compromise patient privacy. However, they are available with the corresponding author and can be obtained on reasonable request.

## Abbreviations

ASC: Adenosquamous carcinoma
CK: Cytokeratin
ICC: Intrahepatic cholangiocarcinoma
CT: Computed tomography
